# Translation and cross-cultural adaptation of the Nepali version of Sexual Interest and Satisfaction Scale (SIS): a cross-sectional study

**DOI:** 10.1186/s41687-024-00816-7

**Published:** 2024-11-29

**Authors:** Govinda Mani Nepal, Sonu Maharjan, Nima Sherpa

**Affiliations:** 1 Department of Physiotherapy, Kathmandu University School of Medical Sciences, Dhulikhel, Kavre Nepal; 2 Star Hospital, Sanepa, Nepal; 3 Department of Physiotherapy, Grande International Hospital, Dhapasi, Nepal

**Keywords:** Spinal cord injury, Sexual Interest and Satisfaction Scale, Nepali, Sexual impairment

## Abstract

**Background and objective:**

Individuals with Spinal Cord Injury (SCI) rank regaining sexual function as their top priority. Sexual dissatisfaction often leads to low self-esteem, reduced intimacy, and relationship difficulties and hence forms an essential part of the rehabilitation process. In Nepal, where people generally hold fairly traditional views about sex and sexual health, a sexual outcome measure that is culturally sensitive is a must for addressing sexual problems. No study has yet been carried out to investigate the impact of SCI on sexual attitudes and satisfaction in Nepal. Therefore, this study aims to translate and cross-culturally adapt the Sexual Interest and Satisfaction Scale (SIS) in the Nepali language.

**Methods:**

(1) Forward translation: Three translators translated the English version of SIS into Nepali. (2) Synthesis: The three translated versions were synthesized into a draft version. (3) Expert committee review: The expert committee meetings were held to reach a consensus for a sensible and applicable pre-final version of SIS. (4) Pre-test: The pre-final version of SIS was tested on 25 individuals with SCI. Their opinions and comprehension were documented for each question, and subsequent modifications were made to form the final version of Nepali SIS (SIS-NP). The internal consistency of SIS-NP was calculated using Cronbach’s alpha.

**Results:**

The translation and cross-cultural adaptation segregated the questions into two divisions: first, which could be answered by everyone irrespective of their sexual partner status, and second, which could only be answered by those with a sexual partner. No changes were made to the original questions; however, essential clarifications and definitions were added. A total of 66 individuals with SCI participated to evaluate internal consistency with a median duration of injury of 9.5 years (IQR = 9.25). Unmarried participants accounted for 40% (n = 27). Internal consistency was found to be 0.74.

**Conclusion:**

SIS was translated to Nepali, adapting standard recommended guidelines. SIS-NP demonstrated adequate internal consistency to be used in SCI.

**Supplementary information:**

The online version contains supplementary material available at 10.1186/s41687-024-00816-7.

## Background

Spinal cord injury (SCI) is an insult to spinal cord, resulting in temporary or permanent change in its normal motor, sensory, or autonomic function [1]. The leading cause of SCI in developed countries is motor vehicle accidents; in non-developed countries, it is falls [2]. This finding of falls as most frequent cause of SCI is similar in Nepalese context too [3]. Reported global incidence of SCI is about 40–80/million/year; in Asia, the incidence of spinal cord injury is 12.06–61.6/million/year [4]. The mean age of individuals sustaining SCI in Nepal is 40 years [3].

One of the consequences of SCI is impairment of sexual function, which results in generally lower sexual satisfaction [5]. Sexual dissatisfaction often leads to poor self-esteem and body image, reduced intimacy, and problems in relationships [6]. Restoring sexual function is considered one of important function by SCI patients within physical domains [7–10] and it is believed that an improvement in sexual function would significantly enhance their overall quality of life [11]. However, there have been reports in which patients with SCI state that sexual rehabilitation is often prioritized less during their rehabilitation [12–14]. To address this issue, there are recommendations to include sexual health rehabilitation in spinal cord injury rehabilitation programs [15].

Sexual knowledge, experience, and attitude in individuals with SCI are also often overlooked in many low and middle-income countries like Nepal [16–18]. This is a concerning issue and highlights the need for a culturally acceptable and reliable measure not only to assess sexual interest after SCI but also to successfully integrate sexual function rehabilitation in SCI rehabilitation programs. The Sexual Interest and Satisfaction Scale (SIS) is one of the few self reported measures designed specifically for individuals with SCI to assess sexual interest and satisfaction before and after injury [19]. It is a 6-item questionnaire covering six primary areas of sexuality, i.e., 1. Sexual desire, 2. Importance of sexuality, 3. General satisfaction with sex life after injury, 4. General satisfaction after injury compared with before the injury, 5. Perceived personal satisfaction, and 6. Self-rated ability to give partner satisfaction. SIS has shown good psychometric properties with internal consistency of 0.96. It has also demonstrated a high correlation with age at injury (Pearson’s r = 0.63) and Quality of Life (QL-VAS method)—Dysfunction rating (Pearson’s r = −0.61), andmoderate correlation with Total QL rating (Pearson’s r = 0.52), QL-Loss of independence (Pearson’s r = −0.49) and QL-Depression (Pearson’s r = −0.45) [19].

In Nepal, where people generally hold fairly traditional views about sex and sexual health, an outcome measure that is culturally sensitive is a must for addressing problems with sexual functioning. A comprehensive and culturally pertinent tool is also crucial in clinical practice and research. Therefore, the primary aim of this study is to translate and cross-culturally adapt SIS in Nepali language in accordance with internationally accepted guidelines [20–22].

## Methods

### Study design and settings

A cross-sectional study design was used, and participants were recruited through snowball sampling given the lack of rehabilitation centers and organizations in Nepal dedicated to SCI [23]. Data were collected either through in-person meetings (following the regulations of social distancing) or online through video calls using online platforms like Facebook and Gmail. Study inclusion criteria included participants who were (1) 18 years or above, (2) diagnosed with spinal cord injury (any level), and (3) duration of injury above one year. All study participants provided written consent (verbal consent for those who could not offer written consent) before completing the questionnaire.

### Translation of SIS into Nepali language

Prior permission was taken from original developers of SIS. Translation and cross-cultural adaptation process were conducted using “Guidelines for the process of cross-cultural adaptation of self-reported measures,” as recommended by Beaton et al. [21]. However, back translation was not performed as reported by Epstein et al. where they concluded that it did not have added benefit compared with expert committee review [22] and retains all important steps as described in best practices [20]. The study was conducted after approval frome Institutional Review Committee (IRC) of Nepal Health Research Council (NHRC), Kathmandu, Nepal (468/2020). The translation process was completed in following four stages:

- **1. Forward translation (FT)**
  Three independent translators translated the English version of SIS into Nepali, resulting in three different Nepali versions (FT1, FT2, and FT3). Among three translators, one was from a non-medical background (a software engineer), and other two were from a medical background (a neuro-physiotherapist and a psychologist); all had Nepali as their first language.
- **2. Synthesis of FT1, FT2, and FT3**
  Principal investigator of this study (GN) synthesized FT1, FT2, and FT3 versions into a single version (FT-123) in presence of a recording observer (SM)—who noted down reasonings provided by the primary investigator for formulating the single version. The guiding principle was based on relevancy, understandability, and accuracy. This single version was then taken to each translator by same recording observer, and their comments were also noted to form the synthesized version.
- **3. Expert committee review**
  The expert committee consisted of three translators, the primary investigator, a university graduate in the medical field, and peer counselor working actively in SCI rehabilitation. The expert committee discussed discrepancies between the original and synthesized versions via teleconference. The final words/phrases chosen from the expert committee were documented. After a consensus was reached among the committee members, a pre-final version of SIS was made.
- **4. Testing of the pre-final version of the measure**
  The pre-final version of SIS was tested on 25 individuals with SCI following Beaton’s guidelines [21]. The participants were additionally asked to fill out an “understanding rating” questionnaire, which consisted of a Likert scale to rate their understanding and a blank space to provide additional suggestions. The Likert scale ranged from scores 1 to 10, where 1 = “not understood at all” and 10 = “completely understood.” Participants were further interviewed with open-ended questions whenever they rated any question below average score (i.e., 5) on the Likert scale. Pre-testing results were presented to the expert committee, and changes were made based on results. Eventually, final version of Nepali-SIS was generated.

### Internal consistency of SIS-NP

We included 66 participants (excluding the pre-testing participants) using convenience sampling to evaluate internal consistency of SIS-NP. Participants were recruited from hostel and rehabilitation center of the Bodhisattvas in Action (BIA) Foundation. BIA is a non-profit distributing social organization that provides handicraft training to generate employment opportunities for people with disability in Nepal [24]. An online accessible format of the SIS-NP was also created and shared among Community Based Rehabilitation (CBR) physiotherapists for data collection. A brief presentation regarding aim of the study, importance of SIS-NP, and procedure of use were given to the CBR physiotherapists before data collection. Informed consent was obtained from all e participants before administering the questionnaire. Measures were taken to maintain confidentiality of data collected.

### Data analysis

Statistical analysis was done using Statistical Package for Social Sciences (SPSS) version 24. Data were assessed for normality using visual (normal distribution curve and Q-Q plot) and statistical methods (Skewness/Kurtosis scores and Shapiro-Wilk’s test) before analyzing psychometric properties of the SIS-NP. Descriptive statistics (mean and standard deviation) were used to summarize continuous data, while frequency and percentage were used for categorical data. Internal consistency was assessed using Cronbach’s alpha. “Cronbach’s α if item deleted” was calculated to determine the homogeneity of items. We considered Cronbach’s alpha values <0.70 as inadequate, 0.70–0.79 as adequate, 0.80–0.89 as good, and >0.90 as excellent [25].

## Results

### Synthesis and expert committee review

Several challenges and concerns emerged during synthesis and adaptation of the SIS scale in Nepali language. Changes were made in following areas:

- **[A] Semantic equivalence:**
  - Several linguistic differences and grammatical preferences for same statement were noted among the translators. Linguistic changes were made to resonate with best translation available during the review.

- **[B] Conceptual equivalence:**
  Several culturally sensitive adaptations were made to the SIS to ensure its relevance and clarity for Nepali participants.
  - Unlike western culture where ‘partner’ might broadly imply a romantic or sexual relationship, in Nepal, ‘sexual partner’ is almost exclusively understood to mean one’s spouse. Therefore, Area no. 6 (i.e., *Self-rated ability to give partner satisfaction*) holds different conceptual meaning in Nepal as the term ‘*partner’* gives impression of a friend rather than a sexual partner. Thus, to avoid ambiguity, the expert committee used the term ‘*youn sathi*’ which translates as’*Sexual partner’* to explicitly represent their spouse. The adapted version read *“youn sathilai santustha dine chyamatako swo-mulyankan,”* meaning “*Self-rated ability to give sexual partner satisfaction.”*

- **[C] Idiomatic equivalence:**
  - We found significant confusion among the translators regarding translation of the word ‘*sexuality’*. The term ‘sexuality’ itself lacks a direct equivalent in Nepali. The suggested translations were *‘youn’ (which means sex)* or *‘kamukta’ (which means eroticism).* Both these words represent a part of sexuality but not sexuality as a whole. Based on the review of the Nepali dictionary and expert consensus, the term *‘younikta’* was selected as the best match for the term ‘sexuality’. However, *‘younikta’* is a broad term related to sex and reproduction without any precise definition. To address this, we incorporated the definition of sexuality (WHO, 2006) at the beginning of the questionnaire to ensure that everyone was at the same level of comprehension. According to WHO, 2016 sexuality is defined as a central aspect of being human throughout life and encompassed sex, gender identities, and roles, sexual orientation, eroticism, pleasure, intimacy, and reproduction. This was simplified and described in Nepali as *“sexuality denotes sex, gender identities, and roles, sexual orientation, eroticism, pleasure, intimacy, and reproduction.”*

### Testing of the pre-final version of the questionnaire

Total of 25 participants were included for pre-testing. Most participants were male (M-19, F-6), with a mean age of 38.88 years. Most participants were educated, and more than half were employed after injury (Table 1).

**Table 1:** Demographic characteristics of pre-testing participants

Variables	Mean or n (%)
Age	38.88
Gender, N (%)	
Male	19 (76%)
Female	6 (24%)
Educational status, N (%)	
Uneducated	3 (12%)
Primary level	2 (8%)
Lower Secondary level	4 (16%)
Secondary level	2 (8%)
Higher Secondary level	4 (16%)
Bachelors level	10 (40%)
Occupation after injury, N (%)	
Unemployed	7 (28%)
Peer counselor for SCI team	5 (20%)
Pension	1 (4%)
Self-employed	4 (16%)
Same job as before the injury	7 (28%)
Working under others	1 (4%)
AIS Level, N (%)	
AIS A	19 (76%)
AIS B	4 (16%)
AIS C	0 (0%)
AIS D	2 (8%)
Level of injury, N (%)	
C3-C5	1 (4%)
T1-T4	1 (4%)
T5-T9	7 (28%)
T10-T12	9 (36%)
L1-L2	7 (28%)
Sexual counseling, N (%)	
Yes	12 (48%)
No	13 (52%)

*Abbreviations AIS* The American Spinal Injury Association (ASIA) impairment scale

The expert committee made multiple changes to pre-final version of the questionnaire based on feedback and pre-testing results. The changes are listed below:

1. Procedure to complete the questionnaire: The most common confusion among participants was the procedure to complete the questionnaire. Participants kept asking if they should put a tick mark on the scale point column or write number in the scale score column. Thus, it was decided to omit the scale score column and insert an instruction at beginning of the questionnaire. The instructions read, *“kripaya tala ka bikalpaharu madhe euta chanauth garnuhos ra yesko cheuma sahi (√) chinna rakhnuhos,”* which translates to *“Please choose one of the following options and place the tick (√) sign next to it”.* Nevertheless, the column was meant for therapist to write the score after interpreting the VAS scale; thus, removing it from a patient-reported scale did not make much difference.
2. Understanding of question no. 5: On the “understanding rating” questionnaire, only question no. 5 (i.e., *Perceived personal satisfaction*) received mean score of less than 5 (See Table 2). In further interview investigating their confusion, participants stated: *“I do not understand what the ability and possibilities to enjoy myself in sex mean.”* After comprehensive discussion, expert committee concluded that confusion was not due to lack of understanding of the question but rather lack of understanding of what it meant to enjoy sexuality on their own. Therefore, it was decided to list probable possibilities of enjoying sexuality on their own at top of the questionnaire as “*youn saririk matra nabhae mansik waa bhawanatmak athawa kunai upakaran, hasthamaithun, kamuk chalchitra adhi bata pani prapta huna sakcha”* which translates to *“Sex is not only physical but mental or emotional potential as well, and it can also be obtained by using any equipment, masturbation, erotic videos, etc.”*
3. Criteria to have a sexual partner before injury: The original SIS required individuals to have sexual partner before injury in order to complete the questionnaire. This eligibility criterion excluded significant portion of SCI population who sustained their injury at young age and were unmarried. As a result, they were unable to use the questionnaire to assess their sexual function. Discussions were held on multiple options to resolve this issue. Finally, the six questions in the original questionnaire were divided into two groups.
  The first group (questions 1, 2, and 6) can be answered by anyone, regardless of their sexual partner status. The second group (questions 3, 4, and 5) is only for those who had a sexual partner before injury. This change makes the questionnaire accessible to all individuals with SCI in Nepal, regardless of marital status or companionship. The two groups were separated by a yes/no question about sexual partner status. Participants were instructed to answer the remaining three questions only if they had a sexual partner.

**Table 2:** Scores given by participants on their “understanding rating” questionnaire

Questions	Number of response	Minimum score	Maximum score	Mean
1. Sexual desire	21	5	10	7.5
2. Importance of sexuality	21	4	10	7
3. General satisfaction with sex life after injury	21	5	10	7.5
4. General satisfaction after injury compared with before injury	21	5	10	7.5
5. Perceived personal satisfaction	21	1	8	4.5
6. Self-rated ability to give partner satisfaction	21	5	10	7.5

4. Use of VAS: When asked for any suggestions to make the scale more straightforward, majority of the participants (61%) expressed that the VAS used in question no. 4 and question no.5 to rate their sexual satisfaction before and after injury, respectively, wereconfusing to them. Participants preferred scale with numbers or a list of options instead of pointing to numberless line. A Verbal Rating Scale (VRS) was thus added, ranging from 0 to 7, where 0 = “very dissatisfying” and 7 = “very satisfying.” A statement explaining that these numbers were in ascending order was also added.
5. Alignment of columns: In the original SIS, second column consisted of questions, and third column consisted of scale points. Participants (42%) expressed that it was complicated to read in columns, and they favored having options directly below the question. Thus, we merged these two columns to form a single second column in the translated version.

### Internal consistency of SIS-NP

A total of 66 individuals with SCI were included in this study. Majority of the participants were young (median = 30.76 years), and 66% of the participants were unemployed or students before the injury. Among those working, high number (n = 8, 12%) were employed in service and sales sector (driver, conductor, security worker, receptionist, cook, and shopkeeper). The baseline characteristics of participants in computing internal consistency is highlighted in Table 3.

**Table 3:** Baseline characteristics of the participants in computing internal consistency

Variable	Median (IQR) or n (%)
Age	30.76 (11.5)
Gender, N (%)	
Male	46 (69.7%)
Female	20 (30.3%)
Educational status, N (%)	
Uneducated	4 (6.3%)
Primary level	7 (10.9%)
Lower Secondary level	7 (10.9%)
Secondary level	10 (15.6%)
Higher Secondary level	21 (32.8%)
Bachelors level	15 (22.7%)
Occupation after injury, N (%)	
Unemployed	27 (40.9%)
Professional	1 (1.5%)
Service and sales worker	8 (12.1%)
Craft and related trades worker	29 (43.9%)
Armed force occupation	1 (1.5%)
AIS Level, N (%)	
AIS A	45 (68.2%)
AIS B	11 (16.7%)
AIS C	10 (15.2%)
Relationship status after injury, N (%)	
Married	27 (40.9%)
Unmarried	39 (59.1%)
Sexual counselling, N (%)	
Yes	36 (54.5%)
No	30 (45.5%)

### Test of reliability

The internal consistency of SIS-NP with all six item’s scores was 0.74. As 17 out of 66 individuals had no sexual partner, they were not eligible to fill the last three items; 49 valid data were used for this calculation.

The internal consistency of only first three item’s scores was 0.77. These three questions could be used by all individuals with SCI (even those without a sexual partner); 66 valid data were used for this calculation. Cronbach Alpha if-item-deleted ranged from 0.49 to 0.73, suggesting that each item had to be retained.

## Discussion

This is a first study to cross-culturally adapt the SIS scale [21, 22]. We made significant cultural changes to make it accessible and practical for large community of individuals with SCI, irrespective of sexual partner status. Our findings have research, clinical, and policy implications.

### Translation and cross-cultural adaptation

Sexuality-related topics are considered taboo in Nepalese societies [26], and pre-marital sexual activities are not socially accepted. Therefore, it is uncommon for people in Nepal to have sexual partners before marriage [27]. The median age for men to get married is 21.6 years, and for women, it is 17.5 years as of 2011 [28]. However, the practice of delayed marriage has increased [29], leading to noticeable increase in median age of marriage [26]. Given that mean age of individuals sustaining SCI is 32.4 years (which is a sexually active group) [30], the criteria of having sexual partner before injury in the original SIS left significant population unable to use the questionnaire. Thus, changes were made to make the questionnaire applicable to all.

Another significant variation in the SIS-NP was preference for VRS over VAS. This result aligns with findings of Pathak et al. [31], where Nepalese population preferred VRS over VAS to rate their pain intensity. They have discussed that the cultural practice in describing quantities and lengths in Nepalese setting differs where they use the terms such as a “handful of rice” and “forearm’s length” which holds true even for our results [31]. While the participants in the study by Pathak et al. were older (median age of 54 years) with no formal education (83%), participants in our study were younger (mean age of 30 years) with some form of formal education (94%). This suggests that cultural factors are more important than age and education in scale preference and needs considerations in future studies [31, 32].

We also found that participants lacked an understanding of possibilities of enjoying sexuality on their own. This was congruent with t findings of Regmi et al., which highlighted lack of knowledge among Nepalese population about sexual and reproductive health [29]. Another study by Stone et al. also pointed out the insufficiency of sex and sexuality education among young individuals in Nepal [33]. This study also emphasizes the need for sex and sexuality education and awareness among the general public and individuals with different disabilities.

### Reliability

We found the internal consistency of SIS-NP to be good. Furthermore, the internal consistency of first three questions also showed good internal consistency. It signifies that first three questions measure common attribute of sexual interest while overall, six questions measure common attribute of sexual interest and satisfaction. The internal consistency in the original article by Siosteen et al. was also calculated to be 0.96, similar to our study [19]. This study supports thefact that SIS-NP is valuable evaluation tool to assess sexual interest and satisfaction levels in individuals with SCI, both with and without a sexual partner in Nepal.

## Strengths and limitations

The strength of this study is applicability of the questionnaire to all adult Nepalese individuals with SCI, irrespective of their sexual partner status. Another strength is that we adapted the questionnaire in a manner which is accessible to all individuals with SCI in Nepal, regardless of marital status or companionship. Furthermore, including participants from different settings like rehabilitation centers, community settings, and hostel settings is an advantage. We have used culturally sensitive sentences and defined all the confusing words like sexuality so that everyone has the same point of view while answering the questions. Data were collected from a renowned rehabilitation and vocational center in Kathmandu, a spinal cord injury hostel for students, and various communities around Kavre. This led to information from diverse perspectives ranging from adolescent to middle age, students to professionals, unmarried to married, and AIS level from A to E, broadly representing the target demographic.

The limitation of this study was its sample size, which was limited due to availability and readiness of the SCI patients to participate. Additionally, the use of snowball sampling and data being primarily collected from a vocational center resulted in a participant pool who were predominantly employed and educated. Female participants were few and outnumbered compared to male participants which may impact the applicability of this measure warrenting further studies. These limitations highlight the need for caution in generalizing the results to the broader SCI population. We recommend future studies to consider inclusiveness in terms of gender, education and employment status and establish the psychometric properties of SIS-NP among individuals with SCI in Nepal.

## Conclusion

SIS was translated to Nepali, adapting standard recommended guidelines. SIS-NP demonstrated adequate internal consistency and can be used in the Nepali population of individuals with SCI. The availability of SIS in Nepali would be the first self-reported sexual questionnaire in Nepali for SCI, which will allow expression of satisfaction level flourishing sexual rehabilitation and form a baseline measurement in the sexual rehabilitation process.

## Electronic supplementary material

Below is the link to the electronic supplementary material.

Supplementary Material 1

## Data Availability

The datasets used and/or analyzed during the current study are available from the corresponding author on reasonable request.

## Abbreviations

AIS: American Spinal Injury Association Impairment Scale
ASIA: American Spinal Injury Association
NHRC: Nepal Health Research Council
NRS: Numeric Rating Scale
QL: Quality of life
SCI: Spinal Cord Injury
SIS: Sexual Interest and Satisfaction Scale
SIS-NP: Nepali version of Sexual Interest and Satisfaction Scale
SPSS: Statistical package for social sciences
VAS: Visual Analogue Scale
VRS: Verbal Rating Scale
WHO: World Health Organization
