# Supplementary material for: Translation and cross-cultural adaptation of the Nepali version of Sexual Interest and Satisfaction Scale (SIS): a cross-sectional study
Source: J Patient Rep Outcomes. 2024 Nov 29;8:138. doi: 10.1186/s41687-024-00816-7 (PMC11607224; doi:10.1186/s41687-024-00816-7)
Supplement: Supplementary file 1 — Supplementary Material 1 [file 41687_2024_816_MOESM1_ESM.pdf]

<u>यौन रुचि र सन्तुष्टि मापन</u>

कृपया तलका विकल्पहरू मध्ये एउटा छनौट गर्नुहोस् र यसको छेउमा सहि (✓) चिन्ह राख्नुहोस्।

(यहाँ यौनिकताले यौन, लैङ्गिक पहिचान, यौनिक अभिमुखीकरण, आनन्द, घनिष्ठता र प्रजननलाई जनाउछ। यौन शारीरिक मात्र नभई मानसिक वा भावनात्मक अथवा कुनै उपकरण, हस्तमैथुन, कामुक चलचित्र आदिबाट पनि प्राप्त हुन सक्छ।)

क्षेत्रहरु	प्रश्नहरू
१. यौन चाहना	तपाईंको यौन चाहना चोट पहिलेको तुलनामा अहिले कस्तो छ?<br>• कुनै चाहना छैन (०)<br>• घटेको छ (१)<br>• कुनै परिवर्तन छैन (२)<br>• बढेको छ (३)
२. यौनिकताको महत्त्व	तपाईलाई यौनिकताको महत्त्व चोट पहिलेको तुलनामा अहिले कस्तो छ?<br>• कुनै चाहना छैन (०)<br>• घटेको छ (१)<br>• कुनै परिवर्तन छैन (२)<br>• बढेको छ (३)
३. व्यक्तिगत सन्तुष्टिको अनुभूति	तपाईंको यौनिकतामा आफु रमाउने क्षमता र सम्भावनाहरू कस्ता छन्?<br>• धैरै असन्तोषजनक (०)<br>• असन्तोषजनक (१)<br>• सन्तोषजनक (२)<br>• धैरै सन्तोषजनक (३)

के तपाइँको यौन साथी हुनुहुन्छ? ☐ छ ☐ छैन

यदि हुनुहुन्छ भने, कृपया निम्न प्रश्नहरुको पनि उत्तर दिनुहोला ।

४. चोटपश्चात यौन जीवनसँगको साधारण सन्तुष्टि *	तपाईंको चोटपछि अधिकांश समयमा यौन साथीसँगको सम्बन्ध कस्तो छ?<br>१ २ ३ ४ ५ ६ ७
५. तुलनात्मक रूपमा चोट लाग्नु अघि र पछिको साधारण यौन सन्तुष्टि **	तपाईंको चोटअघि अधिकांश समयमा यौन साथीसँगको सम्बन्ध कस्तो थियो?<br>१ २ ३ ४ ५ ६ ७
६. यौन साथीलाई सन्तुष्टि दिने क्षमताको स्व. मूल्याङ्कन	तपाईंको यौन साथीलाई यौन चाहना परीपूर्ति गर्न सक्ने तपाईंको क्षमता र सम्भावनाहरू कस्ता छन्?<br>• धैरै असन्तोषजनक (०)<br>• असन्तोषजनक (१)<br>• सन्तोषजनक (२)<br>• धैरै सन्तोषजनक (३)

कुल (०-१८) = ____________

४ क्र.स. र ५ क्र.स. कोलागि रिपोर्ट गरिएको प्राप्तांक ०-३ दायरा बीचको हुँदैन। रिपोर्ट गरिएको प्राप्तांक लिनुहोस् र समग्र प्राप्तांक (०-१८) को लागी उचित मापन प्राप्तांक (०-३) पत्ता लगाउन तलका तालिकाहरूसँग तुलना गर्नुहोस्।

* समग्र प्राप्तांकको लागी मिलाउदा (४ क्र.स):

VRS प्राप्तांकको दायरा:	समग्र प्राप्तांक (०-३):
१	०
२ - ३	१
४ - ५	२
६ - ७	३

** समग्र प्राप्तांकको लागी मिलाउदा (५ क्र.स.):

VRS भिन्नताको प्राप्तांकको दायरा::	समग्र प्राप्तांक (०-३):
<-२	०
-२ वा -१	१
०	२
>०	३

० - १८ को अन्तिम अंक प्राप्त गर्न प्रत्येक कोटिबाट प्राप्तांकहरू सँगै जोड्नुहोस्।
